# Impact of COVID-19 Governmental Restrictions on Emergency General Surgery Operative Volume and Severity

**DOI:** 10.1177/00031348211011113

**Published:** 2021-04-16

**Authors:** Sarah Lund, Taleen MacArthur, Marianna Martini Fischmann, Justin Maroun, Johnny Dang, James R. Markos, Martin Zielinski, Daniel Stephens

**Affiliations:** 1 Department of General Surgery, 4352 Mayo Clinic Rochester, MN, USA; 2 Alix School of Medicine, 4352 Mayo Clinic Rochester, MN, USA; 3 Department of Trauma, Critical Care, and General Surgery, 4352 Mayo Clinic Rochester, MN, USA

**Keywords:** acute care surgery, trauma acute care, operative volume, COVID-19, emergency general surgery

## Abstract

**Background:**

To describe the effect of the COVID-19 pandemic on emergency general surgery operative volumes during governmental shutdowns secondary to the pandemic and characterize differences in disease severity, morbidity, and mortality during this time compared to previous years.

**Methods:**

This retrospective cohort study compares patients who underwent emergency general surgery operations at a tertiary hospital from March 1st to May 31st of 2020 to 2019. Average emergent cases per day were analyzed, comparing identical date ranges between 2020 (pandemic group) and 2019 (control group). Secondary analysis was performed analyzing disease severity, morbidity, and mortality.

**Results:**

From March 1st to May 31st, 2020, 2.5 emergency general surgery operations were performed on average daily compared to 3.0 operations on average daily in 2019, a significant decrease (*P* = .03). No significant difference was found in presenting disease severity, morbidity, or mortality between the pandemic and control groups.

**Discussion:**

This study demonstrates a decrease of 65% in emergency general surgery operations during governmental restrictions secondary to the COVID-19 pandemic. This decrease in operations was not associated with worse disease severity, morbidity, or mortality.

## Introduction

In an attempt to control the spread of the novel coronavirus 2019 (COVID-19), governments worldwide have implemented various restrictions and containment strategies. In some areas of the United States, this included limiting elective surgeries. These policies had the intended impact of decreasing elective surgical burden, but the impact on emergent surgical operative volume is unknown. As international data suggest, patients may have been dissuaded from seeking care in the emergency department (ED) due to concern about exposure to COVID-19.^1^ Several studies have shown significant decreases in ED visits, trauma admissions, and urgent medical admissions (ie acute coronary syndrome) during governmental restrictions.^2-4^ Given these trends, we aimed to assess if pandemic-related governmental restrictions also decreased emergency general surgery operative volume at our institution, which is a quaternary academic hospital.

## Methods

This retrospective cohort study was approved by an institutional COVID-19 Research Task Force and deemed exempt by Mayo Clinic’s Institutional Review Board. The volume of emergency general surgery operations at Mayo Clinic, Rochester, was compared between 2 time periods: March 1st to May 31st, 2020 (pandemic period) vs March 1st to May 31st, 2019 (control period). These 2 time periods were analyzed based on the timeline of governmental shutdowns in Minnesota. In Minnesota, closure of nonessential businesses was mandated by the governor’s executive order on March 16th, 2020, and a shelter-in-place order was in effect from March 25th, 2020 to May 18th, 2020, during which time people were mandated to stay home, with the exception of essential activities such as obtaining food or seeking medical attention. Furthermore, Mayo Clinic was mandated, along with other hospitals in Minnesota, to postpone elective procedures from March 17th to May 1st Therefore, the time periods selected included at least 2 weeks before and after shelter-in-place orders and postponement of elective procedures. The control period was similar in duration, during the same time of year, and was selected during the previous year (a year unaffected by a global pandemic). To better understand the trend in operative volume, the same 2-week periods within the pandemic and control periods were compared.

Our emergency general surgery practice consistently performs approximately 3200 operations per year, which has been stable year to year. The current study adheres to the Strengthening the Reporting of Observational Studies in Epidemiology (STROBE)^5^ guidelines for reporting observational studies. Patients were included in chart review if they underwent emergency general surgery during the specified time frames. We reviewed the electronic medical record for several variables including patient demographics (age, sex, and race), vital signs upon initial hospital presentation, Acute Physiology, and Chronic Health Evaluation (APACHE) II score, American Society of Anesthesiologists (ASA) score, operative American Association for the Surgery of Trauma (AAST) grade,^6^ indication for operation, and type of procedure. For outcomes, we assessed the following factors: intensive care unit (ICU) admission, hospital length of stay, intra- and postoperative complications, in-hospital mortality, and the number of patients who required reoperations.

Quantitative variables were reported as means and ranges, while categorical variables were reported as frequency counts and percentages. These variables were compared between the control and pandemic periods; univariate analysis was performed with X^2^ for categorical variables and Student’s t-test for quantitative variables. Significance was determined with α < .05, and all hypothesis tests were two-sided. Sample size calculation was performed using Student’s t-test for testing between control and pandemic groups. A study with an effect size of .3 and a power of 90% required a total sample of 470 (235 in each group) to find a significance at α = .05. A second sample size calculation was performed using X^2^ test. For this power analysis, a study with an effect size of .3 and power of 95% required a total sample of 220 to find significance at α = .05.

## Results

A total of 508 emergency general surgery operations occurred in the pandemic and control periods, with 279 emergent operations performed in the control period and 229 emergent procedures performed in the pandemic period. Table 1 provides a comparison of patient characteristics and selected outcomes between the control and the pandemic groups. The only significant difference observed in patient characteristics was in patient ASA score, which was 2.6 for the control group and 2.8 for the pandemic group (*P* = .03). No significant difference was observed for any other patient characteristics, including demographics, patient vitals upon initial hospital presentation, APACHE score, frequency of conversion to open approach, perioperative ICU admission, AAST grade (commonly used and validated in emergency general surgery to evaluate severity of pathology), intraoperative or postoperative complications, rate or number of reoperations, length of hospital stay, or mortality.

**Table 1.** Comparison of Emergency General Surgery Patient Characteristics and Outcomes in Pandemic Compared to Control Periods. (*, *P* < .05).

		2019	2020	*P*-value
Demographics	Age, mean (SD)	56.7 (18.3)	59.3 (18)	.12
	Gender (% female)	158 (57%)	127 (56%)	.83
	Race (% white)	251 (90%)	200 (87%)	.60
Type of operation				.80
	Laparoscopic	165 (59%)	139 (61%)	
	Open	88 (32%)	78 (34%)	
	Conversion to open	26 (9%)	12 (5%)	
Illness severity	APACHE score, mean (SD)	4.7 (1.4)	4.7 (1.4)	.47
	ASA score, mean (SD)	2.6 (.9)	2.8 (.9)	.03*
	AAST grade, mean (SD)	1.95 (1.2)	2.1 (1.2)	.33
	Tachycardia on admission	54 (19%)	48 (21%)	.99
	Hypotension on admission	17 (6%)	13 (6%)	.99
	ICU admission	65 (23%)	53 (23%)	.99
COVID-19-positive patients		N/A	1 (1%)	
Complications	Intraoperative	11 (4%)	8 (4%)	.99
	Postoperative	69 (25%)	49 (21%)	.94
Length of stay, mean (SD)		6.8 (11.4)	6.7 (12)	.92
Mortality		12 (4%)	12 (5%)	.99

Abbreviations: ASA, American Society of Anesthesiologists; AAST, American Association for the Surgery of Trauma; ICU, intensive care unit.

When comparing total emergent case volumes between the control and pandemic periods, the total emergency general surgery operations performed in the pandemic period was significantly less at 2.5 operations per day on average compared to the control period at 3 operations per day (*P* = .03) (Table 2). Furthermore, 65% less emergency general surgery operations were performed during the early weeks of the governmental restrictions, March 15th to April 25th, in 2020 compared to the same period in 2019 (Table 2). A range of operations were performed, with laparoscopic cholecystectomies and appendectomies making up the majority of the operations performed in both the pandemic and control periods. There was no significant difference between the pandemic and control periods with regard to frequency of operative indications or type of operation performed.

**Table 2.** Comparison of Emergency General Surgery Operation Volume Between Pandemic and Control Periods. (*, *P* < .05).

Date range	2019 no. of operations per day (mean [SD])	2020 no. of operations per day (mean [SD])	*P*-value
Entire period (3/1-5/31)	3.0 (1.8)	2.5 (1.6)	.03*
3/1-3/14	2.2 (1.5)	3.1 (1.8)	.15
3/15-3/28	3.7 (1.9)	1.8 (1.2)	.004*
3/29-4/11	4.4 (2.3)	1.5 (.5)	< .001*
4/12-4/25	2.6 (1.4)	1.6 (1.3)	.04*
4/26-5/9	2.9 (1.3)	3.4 (1.7)	.46
5/10-5/23	2.5 (1.0)	3.0 (1.6)	.33
5/24-5/31	2.4 (2.3)	3.1 (1.2)	.54

## Discussion

Finding a decrease in emergency general surgery operations performed during the pandemic period, while surprising, was in line with our hypothesis. Other international studies have demonstrated a decrease anywhere from 40 to 66% in emergency general surgery cases during the early pandemic period.^7-10^ The evident decrease in emergency general surgery operations during the pandemic shutdown seen in this study and internationally has led to this study’s hypothesis that patients may be avoiding or delaying emergency cares due to concern of COVID-19 exposure or fear of overstretching hospital resources. A retrospective review from the United Kingdom reported that patient’s duration of symptoms prior to presentation was significantly longer in the pandemic period; however, their New Early Warning 2 (NEWS2) score and inflammatory markers were not significantly higher.^11^ Another study from Spain further demonstrated a longer duration in symptoms prior to presentation and also demonstrated higher postoperative morbidity; however, their reoperation and mortality rates were not different.^12^ Interestingly, despite the fact that patients may have delayed care, we did not see a worsening of patient postoperative outcomes or illness severity during the early pandemic period or during the 1 month period after emergency general surgery operative volume returned to baseline.

As of November 2020, the United States has entered another surge of COVID-19 cases, with daily case numbers nearing 200 000. The current wave of the pandemic has had a far wider reach than what was observed in the spring of 2020 and has devastated communities across the nation. As many states once again increase restrictions to attempt to curb the spread of the virus, it is essential that we look back at our experiences in the spring to help understand what is to come over the next several months. This way, we may have well-informed, data-driven conversations when counseling patients. Based on our institution’s observations, it appears that our patient population likely did avoid seeking emergency surgical care during the governmental restrictions in the spring, but that this fortunately did not impact patient outcomes.
